# Structure of Sewage Sludge-Clay Multiscale Composite Particles to Control the Mechanism of SO_2_ and H_2_S Gas Release

**DOI:** 10.3390/ma15051855

**Published:** 2022-03-02

**Authors:** Haihong Fan, Lin Li, Zhou Li, Shuo Shang

**Affiliations:** College of Materials Science and Engineering, Xi’an University of Architecture and Technology, Xi’an 710055, China; lilylee0823@163.com (L.L.); leezu1997@163.com (Z.L.); ss08152021@163.com (S.S.)

**Keywords:** sewage sludge, clay, multiscale composite particles, H_2_S, SO_2_, inhibition mechanism

## Abstract

In order to address the problem of sulfur gas and other odors released in the process of using sewage sludge as a construction material, this study prepared multiscale composite particles with a “large scale-medium scale-small scale-micro scale” structure by mixing sludge with silica-alumina building materials. Analysis of the structural changes formed by the internal gas of composite particles due to diffusion at different temperatures and a study of the characteristics of SO_2_ and H_2_S release from composite particles were conducted, as well as being compared with the release characteristics of pure sludge, which clarified the mechanism of controlling sulfur-containing-gas release from composite particles. The results showed that compared with pure sludge, the sludge-clay multiscale composite particles were able to reduce the release of SO_2_ and H_2_S up to 90% and 91%, and the release temperatures of SO_2_ and H_2_S were increased to 120 °C and 80 °C, respectively. Meanwhile, the special structure of the sludge-clay multiscale composite particles and the clay composition are the main factors that hinder the diffusion of sludge pyrolysis gases. Additionally, there are three layers of “gray surface layer-black mixed layer-dark gray spherical core” formed inside the composite particles, which is the apparent manifestation of the diffusion of volatile gases. This study provides theoretical support for the application of multiscale composite particle inhibition of odor-release technology in industrial production.

## 1. Introduction

With the acceleration of urbanization and the increase in people’s awareness of environmental protection, the capacity of urban wastewater treatment and the host of sludge produced have increased dramatically. In terms of composition, sewage sludge (SS) is a mixture of organic matter, sand, mineral impurities, water, and other components. It contains a large number of organic acids, inorganic acids, phenols, mineral particles, pathogenic bacteria, heavy metals, and other harmful substances, as well as bacterial flora and useful compounds (carbon, nitrogen and phosphorus compounds) [1,2]. If not properly treated, it can have substantial impact on the environment, soil, and people’s daily life [3]. According to statistics, the volume of dry sludge in China reached 5.61 million tons in 2018 [4], sludge production has exceeded 60 million tons in 2019 (based on 80% water content), and the annual production of sludge is expected to exceed 90 million tons in 2025 [5]. At present, the utilization of construction materials is an important method of sludge treatment and disposal, which is cheaper and more widely used than other treatment and disposal methods. Meanwhile, it can reduce the impact on soil, groundwater, and environment in the process of treatment and application. 

The utilization of sewage sludge construction materials is mainly based on drying the sludge, thoroughly mixing it with silica-alumina raw materials such as clay, and preparing it into cement [6], bricks [7], and ceramic granules [8] after heating or firing and other processes, in which the sludge releases odorous gases, causing a large impact on the environment. Clay is composed of a variety of hydrated silicates and certain amounts of alumina, alkali metal oxides, and alkaline earth metal oxides, possessing a well-developed pore system, with a large specific surface area and good adsorption properties, and has gained wide and intensive applications in the area of adsorptive separation and environmental treatment [9,10]. Zheng et al. [11], showed that firing pretreatment of sludge removed 52.8% of S from sludge but slowed down the thermal rate of sludge, increasing the cost of pyrolysis. Wang et al. [12], studied the effect of dewatering conditioners on N/S/Cl contaminants in sludge, and it was found that SO_4_^2−^ in the conditioner enhances the release of SO_2_ but inhibits the release of N-containing contaminants. Liu et al. [13], used chemical conditioners: polyacrylamide (PAM), H_2_SO_4_, and CaO to inhibit the pyrolysis process in sludge sulfur-containing gas release, and revealed that PMA and H_2_SO_4_ increase SO_2_ and H_2_S emissions, while CaO can reduce sulfur-containing gas emissions by producing calcium sulfate. Cheng et al. [14] added alkalis (KOH and NaOH) to the sludge and discovered that they inhibit the release of H_2_S but accelerate the release of SO_2_ under low temperature. As mentioned above, although these methods have some inhibitory effect on sludge odor release, there is still some odor gas from escaping and the cost of odor treatment is high. In contrast, it is more suitable to use clay as a material to inhibit the release of sulfur-containing gas from sludge, because clay not only has the characteristics of low cost and simple production process, but it also has developed applications in industrial gas adsorption products, and it is more often used as an adsorbent pelletizing binder formation. Milica et al. [15] used materials such as sludge, fly ash, and landfill ash and mixed them into a representative heavy clay material. It was found that the samples with 50% fly ash and landfill ash additions had the greatest plasticity. In an aqueous solution, bentonite behaves as a binder to bridge mineral particles [16], and bentonite has also been proven to be the most effective binder [16,17]. Therefore, it is very suitable to use clay to prepare sludge-clay multiscale composite particles (SS-C composite particles) for sludge odor adsorption.

The present study made multiscale composite particles with a specific hierarchical structure of sludge mixed with clay and achieved the regulation of harmful gas release by using the special structure of this kind of particles and the adsorption effect of clay. It aimed to prepare sewage sludge-clay composite particles to control the release of odorous gases from sludge, enable the sludge to be consumed in large quantities, and to solve the environmental pollution problems caused during the utilization of building materials from sludge.

## 2. Materials and Methods

### 2.1. Materials

The dewatered sewage sludge used in this study was sampled from the Xi’an wastewater treatment plant. First, the wet sludge was dried in an oven at 105 °C for 24 h to achieve a constant weight; then, the dry sludge was crushed and sieved to about 105 μm, and the dry clay was crushed and sieved to about 20 μm, so as to obtain dry sludge and dry clay fine powder. Meanwhile, they were stored separately in airtight containers for spare. Finally, the SS-C composite particles were prepared according to the multiscale particle preparation method.

**Figure 1:**
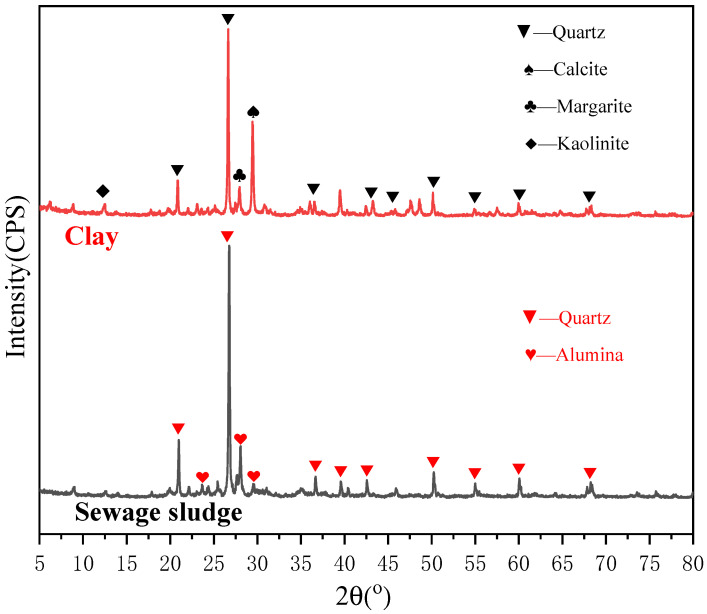
XRD patterns of SS and clay.

The results of the proximate analysis of the SS are presented in Table 1. Table 2 and Table 3 showed the chemical compositions of sludge and clay, It can be seen that the sludge contains a large amount of silica-aluminous components and also contains some Fe and P-containing substances. On the other hand, clay contains a lot of calcium-containing substances in addition to silica-alumina components. Figure 1 is the XRD pattern of SS and clay, where it can be seen that the primary mineral phases of sewage sludge are quartz and alumina, and the primary mineral phases of clay are quartz, calcite, margarite, and kaolinite. Therefore, SS-C composite particles contain a certain amount of SiO_2_, Al_2_O_3_, and CaCO_3_, etc.

### 2.2. Methods

#### 2.2.1. TG

The TG characteristics of sewage sludge were tested on a TGA-DSC-1600 thermal analyzer (Mettler-Toledo, Zurich, Switzerland). The protective atmosphere was nitrogen (N_2_, 99.999%), the N_2_ flow rate was 20 mL/min, the experiments were performed using a 70 μL alumina crucible, and the sample weight was about 15 ± 0.1 mg. The test temperature was 50~1100 °C, and the heating rate was 10 °C/min. The mass loss was calculated from the weight difference of each sample before and after the experiment [18].

#### 2.2.2. Sewage Sludge-Clay Multiscale Composite Particles with Sulfur Gas Online Monitoring Test

**Figure 2:**
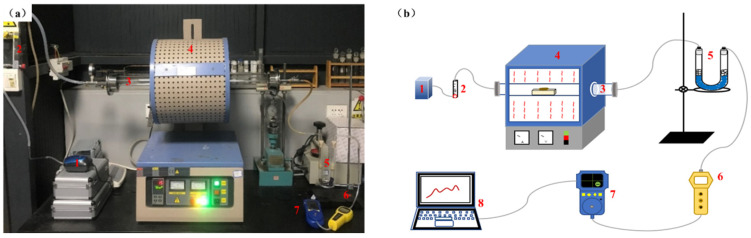
Sulfur-containing gas detection test bench: (**a**) Combustion and sulfur-containing-gas detection device diagram; (**b**) combustion and sulfur gas detection diagram; 1-air pump; 2-rotor flow meter; 3-quartz tube; 4-tube furnace; 5-U-tube; 6-suction pump; 7-gas detector; 8-computer.

This experiment used a QSH-VTF-1200T tube furnace (Shanghai Quanshuo, Shanghai, China) to heat the sample, and a HFP-0401 (C) portable gas detector (Xi’an Huafan, Xi’an, China) for the online testing of the gaseous products from sample decomposition. Meanwhile, a HUIKE-K2 air pump (Shenzhen HUIKE, Shenzhen, China) provides the combustion atmosphere and a HFP-BX suction pump (Xi’an Huafan, Xi’an, China) provides the gas detector with a constant flow of odorous gas. Figure 2 shows the sulfur-containing gas detection test bench, where the gases are identified with the air pump → rotameter → quartz reactor → U-tube → suction pump → gas detector. Among them, the air pump played the role of providing combustion atmosphere and transporting decomposition products, the sample was placed in an alumina crucible and then heated in a quartz tube, and the U-tube was added with quartz cotton and silica-gel desiccant, which played the roles of filtering tar and drying gas, respectively. Air was chosen as the carrier gas for the experiments, and the flow rate of both air and pumping was 205 mL/min.

## 3. Results and Discussion

### 3.1. TG-DTG Analysis of Sludge

**Figure 3:**
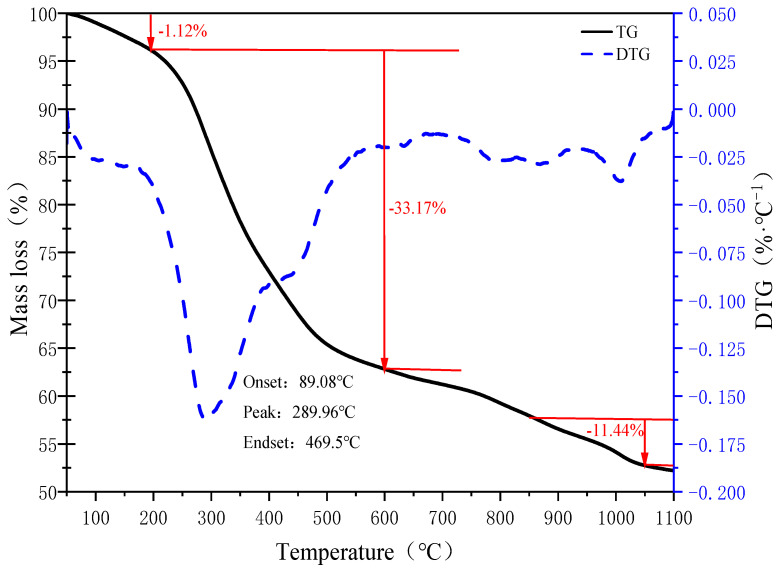
TG-DTG curves of SS.

The TG-DTG curves of SS are shown in Figure 3. The mass loss process can be divided into three stages: (1) The drying and water evaporation stage from 50 °C to 200 °C, at this stage, the SS sample absorbs heating slowly and mainly loses mass due to water evaporation, and the mass loss is only 1.12%; (2) the pyrolysis and charring stages are from 200 °C to 600 °C, and among 200~400 °C is the pyrolysis stage, which is mainly associated with mass loss caused by the decomposition of unstable proteins and volatilization of organic matter [19,20]. In addition, 400~600 °C is the charring stage, with a mass loss of 11.44%, and the organic matter (aliphatic-S and aromatic-S [21]) in this section of SS will be slowly pyrolyzed until charring into char. Above all, the mass loss at this stage is the largest and the maximum mass loss is at temperature point 290 °C; (3) 600~1100 °C is the stage of decomposition of refractory organic and inorganic substances, in which the mass loss from 600~950 °C is due to the decomposition of residual volatile organic substances, sulfate and carbonate [21,22] in SS, while 950~1100 °C is associated with mass reduction because of the decomposition of char. 

In summary, It is known that the mass loss of sludge is maximum in the temperature range of 200 °C to 600 °C, so the decomposition of organic matter also accelerates the release of gas. It is reported in other literature that the mass loss of sludge at 200~600 °C can reach 41.7~51.8% [19,23,24,25]. Therefore, regulating the gas release process in the temperature range of 200 °C to 600 °C is the key to reducing the release of harmful gases during sludge drying. 

### 3.2. Preparation of Sludge-Clay Multiscale Composite Particles

The “multiscale composite particle preparation method” is similar to the classification of particle size [26,27]. For example, particles are usually broadly classified by particle size as “nanoparticles (1–100 nm)”, “ultrafine particles (0.1–1 μm)”, “fine particles (1–100 μm)”, “coarse particles (100–1000 μm)”, etc. However, the particle size ranges of the above classifications may vary in different industries. Therefore, this study has given a new definition for the structure of SS-C composite particles.

**Figure 4:**
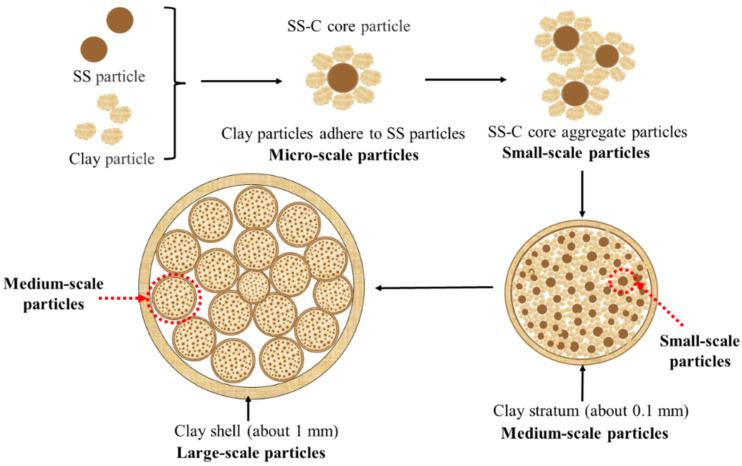
Schematic structure of sewage sludge-clay multiscale composite particles (SS-C composite particles).

The structure of the SS-C composite particles is shown in Figure 4. On the whole, the composite particles are spherical in shape and belong to the large-scale particles with diameters of about 15–20 mm. These are divided into internal and external layers; the outermost layer is a thin clay layer made of clay micropowder, closely accumulated, while the inner layer is a particle cluster made of multiple small-scale composite particles with diameters of about 1 mm adhered to each other, and this particle cluster is defined as medium-scale particles. Meanwhile, the small-scale particles are also composed of an inner and outer core-shell structure, with the outer layer consisting of an accumulation of clay micronutrients and the inner layer consisting of multiple smaller-scale particles. However, these smaller scale particles are composed of smaller microscale core-shell structures of sewage sludge-clay cores. Therefore, the SS-C composite particle refers to a multiscale composite particle with a “macro-scale-mesoscale-micro-scale” structure.

### 3.3. Changes in the Internal Structure of Sewage Sludge-Clay Multiscale Composite Particles during Heating

This study aimed to analyze the mechanism of the sewage sludge-clay multiscale composite particles how to control the release of SO_2_ and H_2_S. Hence, according to the sludge and clay ratios listed in Table 4 (that is, in ratios 1#–6#: 1 g of sludge and 5 g, 10 g, 15 g, 20 g, 25 g and 30 g of clay separately), SS-C composite particles with different ratios were made and heated at different temperatures, and the firing regime was 0–200 °C, 300 °C, 400 °C, 500 °C, 600 °C, 700 °C, 800 °C, 900 °C, 1000 °C, and 1100 °C with a continuous heating rate of 10 °C/min, holding for 5 min, cooling to room temperature and then removed. Finally dissected to observe the cross-sectional changes. The results are shown in Table 5. 

**Figure 5:**
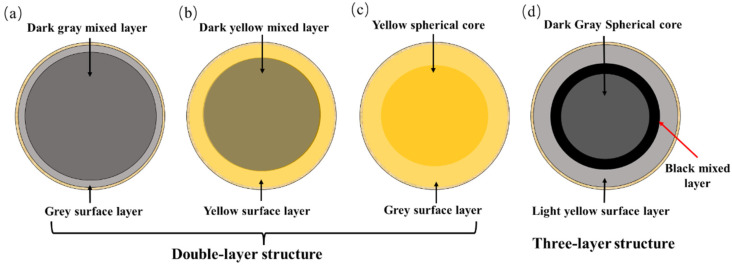
Schematic diagram of the internal structural changes of SS-C composite particles. (**a**) Gray surface layer-dark gray mixed layer, (**b**) yellow surface layer-dark yellow mixed layer, (**c**) light yellow surface layer-yellow spherical core, (**d**) gray surface layer-black mixed layer-dark gray spherical core.

As seen in Table 5, in the temperature range of 105~300 °C, the interior of the spherical SS-C composite particles exhibited a double-layer structure of “gray surface layer-dark gray mixed layer” from the outside to the inside (Figure 5a). With the increase in the proportion of clay, the color of the dark gray core kept becoming shallower. During the temperature range of 400~600 °C, when the sludge-clay ratio was 1:5, 1:10, and 1:15, the inner part of the SS-C composite particles presented a three-layer structure of “gray surface layer-black mixed layer-dark gray spherical core” from outside to inside (Figure 5d), and when the ratio was 1:20, 1:25, and 1:30, the inner part of the SS-C composite particles was “yellow surface layer-dark gray spherical core”. The two-layer structure is of “yellow surface layer-dark yellow mixed layer” (Figure 5b), but the mixed layer is darker than at low temperature. However, the color of the mixed layer was darker than that at low temperature. When the temperature was higher than 700 °C, only the SS-C composite particles with the sludge-clay ratio of 1:5 showed the bilayer structure of “light yellow surface layer-yellow spherical core” (Figure 5c), and the internal color of the particles in other ratios was basically uniform. 

The change in color inside the SS-C composite particles is a macroscopic expression of the outcome of the sludge pyrolysis reaction, in which more biochar [28] or VOCs containing carbon are retained by pyrolysis, and the color becomes darker. The change in color at different temperatures was analyzed to speculate on the pyrolysis process of sludge inside the composite particles.

The surface layer of SS-C composite particles is a clay layer, and under low temperature, carbon-containing gas produced by internal sludge pyrolysis diffuses outward and is trapped, partially due to the adsorption of clay, which has a low concentration and presents a gray color. However, clay layer minerals undergo decomposition-sintering and other reactions in the high-temperature environment above 700 °C and present a yellow color.

The SS-C composite particles consist of a mixture of multiscale composite particles made of sludge and clay powder in a certain ratio, and during the process of sludge pyrolysis, the adsorption of clay and the resistance of particles of different scales cause a large amount of biochar or carbonaceous organic materials diffused outside the particles to be retained here, and the more the retained materials, the darker the color. The three-layer structure of “gray surface layer-black mixed layer-dark gray spherical core” in the temperature range of 40~600 °C in Table 5 indicates that the outwardly diffused carbon-containing materials gather in the area of “black mixed layer” in this temperature range. When the temperature is higher than 700 °C, there is no stratification inside the composite particles, which means that all the carbon-containing materials in the sludge have been decomposed. 

As can be seen from Table 5, the preparation of sludge and clay into multiscale composite particles at a drying temperature that was equal to or lower than 600 °C could effectively control the rate of pyrolysis of organic matter in sludge and reduce the amount of gas released. 

### 3.4. Characteristics of SO_2_ and H_2_S Release from Sewage Sludge-Clay Multiscale Composite Particles

In industrial applications, a 1:5 SS-C composite particle is equivalent to containing 50% wet sludge and 50% dry clay, which in turn reduces the plasticity of the particle [15]. Therefore, it is significant to consider 1:5 SS-C composite particles as a research object.

**Figure 6:**
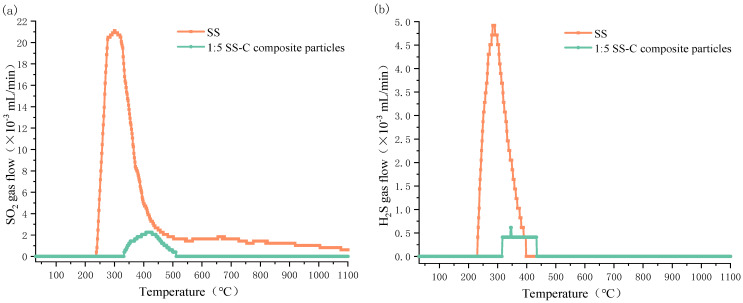
Release of SO_2_ and H_2_S gases from SS and 1:5 SS-C composite particles at different temperatures; (**a**) SO_2_; (**b**) H_2_S.

Different temperatures of SO_2_ and H_2_S release from sludge and 1:5 SS-C composite particles are shown in Figure 6. It is seen that compared with heating 1 g sludge and composite particles containing 1 g sludge, the release of SO_2_ and H_2_S from sludge is much larger than that from composite particles, whereas the temperature at which SS-C composite particles start to release SO_2_ and H_2_S is significantly higher than that from sludge.

From the analysis of Figure 6a, it is clear that SO_2_ gas is released in large quantities from sludge in the temperature range of 250 °C to 450 °C, while it releases H_2_S gas in large quantities from 250 °C to 400 °C. The peaks of release occurred at 300 °C and 320 °C, and at the same time, the corresponding maximum release amounts reached 21 × 10^−3^ mL/min and 4.8 × 10^−3^ mL/min.

As can be seen from the analysis in Figure 6b, the temperature ranges for the massive release of SO_2_ and H_2_S gases from SS-C composite particles are, respectively 330~500 °C and 320~450 °C, which are higher than the minimum temperatures for the massive release of both gases from pure sludge by 80 °C and 70 °C, and at the same time, there are peaks at 420 °C and 400 °C, which are higher than that of sludge by 120 °C and 80 °C, accordingly, and the peaks of release are 2.1 × 10^−3^ mL/min and 0.4 × 10^−3^ mL/min, which are 90% and 91% lower than that of sludge. 

### 3.5. Mechanism of Sulfur-Containing-Gas Release from Sewage Sludge-Clay Multiscale Composite Particles

Comparing the internal structure of the SS-C composite particles (Table 5) and the amount of sulfur gas released at different temperatures (Figure 6), it can be seen that there is a coincidence between the temperature when the SS-C composite particles start to show a two-layer structure of “gray surface layer-dark gray mixed layer” and the temperature point at which SO_2_ and H_2_S gases are released. Furthermore, the temperature point of the beginning of the three-layer structure of “gray surface layer-black mixed layer-dark gray spherical core” corresponds to the temperature point where the maximum amount of SO_2_ and H_2_S are released. However, when the inner part of the SS-C composite particles became a double-layer structure of “yellow surface layer and dark yellow mixed layer”, the two gases were not detected in the released gas. Therefore, it can be inferred that the “large scale-medium scale-small scale-micro scale” structure of the SS-C composite particles inhibit the release of both SO_2_ and H_2_S sulfur-containing gases.

**Figure 7:**
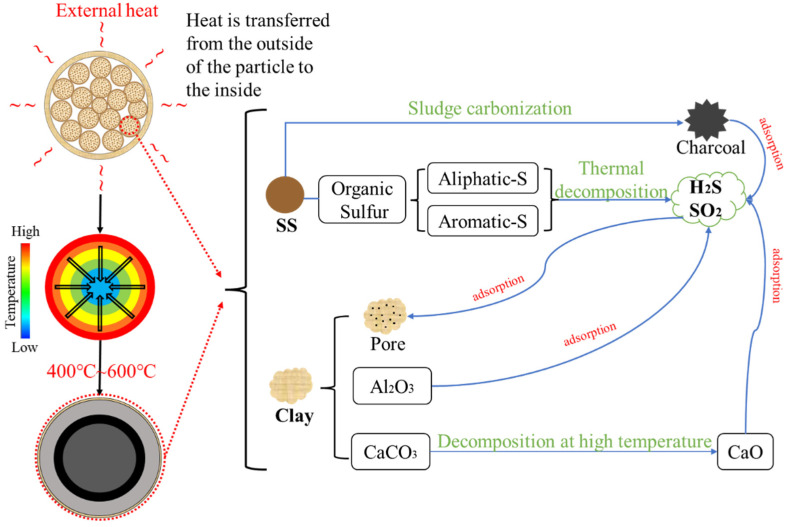
Inhibition of sulfur-containing-gas release process by SS-C composite particles.

As shown in Figure 7, the reason why SS-C composite particles can inhibit the release of sulfur-containing gases is that sulfur in sludge is mainly in the form of organic matter, and the sulfur-containing-gas release is due to the decomposition of organic sulfur (aliphatic-S and aromatic-S, etc.). With the increase in temperature, the organic matter in the sludge will diffuse by thermal decomposition, and the semi-volatile organic matter will continuously diffuse out of the shell because the clay shell layer has no organic matter while the mixed layer has a high concentration of organic matter. In the process of diffusion, H_2_S and SO_2_ are adsorbed in the pores of clay to reduce the release of sulfur-containing gases, and the multiscale structure also blocks the escape of gases and the transfer of organic matter. Qie [29] et al. found that SO_2_ could be adsorbed by different layers of pores, but the highest amount of SO_2_ was adsorbed by micropores, 33.0 mg/g; Gasquet [30] et al. found that H_2_S could also be adsorbed by the micropores of activated carbon; Yang [31] et al. The mechanism of H_2_S and SO_2_ removal from 13X molecular sieves (with porous structure) is an adsorption-redox process, in which H_2_S is oxidized to singlet sulfur and SO_2_ is oxidized to sulfuric acid attached to the pores. Meanwhile, the alkaline component and CaO react together to adsorb SO_2_ and H_2_S as adsorbents [32].Therefore, the organic matter accumulates in the black mixed-layer area, but with the increase in temperature, the organic matter accumulated in the black mixed-layer area will be decomposed and diffused outward.

## 4. Conclusions

In this study, sewage sludge-clay multiscale composite particles (SS-C composite particles) with “large scale-medium scale-small scale-micro scale” structure were prepared from sewage sludge and clay. The structural changes in the multiscale composite particles due to diffusion of internal gases at different temperatures and the release characteristics of SO_2_ and H_2_S were also investigated. In addition, compared with the release characteristics of sludge, the mechanism of multiscale composite particles to control the release of sulfur-containing gases was clarified. It is concluded from the experimental results as follows:

(1) The mass loss in the temperature range of 200 °C to 600 °C is the largest in the sludge-drying process, and the mass loss amounts to 33.17%.
(2) The special structure of sewage sludge-clay multiscale composite particles and the composition of clay can prevent the diffusion of sludge pyrolysis gas. Furthermore, it can form a double-layer structure of “gray surface layer-dark gray mixed layer”, “gray surface layer-black mixed layer-dark gray spherical core”, and “light yellow surface layer-yellow spherical core” within the composite particles at different temperature intervals.
(3) The “large scale-medium scale-small scale-micro scale” structure of the sewage sludge-clay multiscale composite particles can inhibit the sulfur gas release of SO_2_ and H_2_S.
(4) Sewage sludge-clay multiscale composite particles are also 80 °C and 70 °C higher than the minimum temperature at which SO_2_ and H_2_S gas are, respectively, released from pure sludge. Moreover, the peak temperature point is also 120 °C and 80 °C higher than that of pure sludge, and the maximum release is 90% and 91% lower than that of pure sludge.
(5) The double-layer structure of “gray surface layer-dark gray mixed layer” and the three-layer structure of “gray surface layer-black mixed layer-dark gray spherical core” formed by sewage sludge-clay multiscale composite particles are the best internal structures to control the release of sulfur-containing gases.

On the one hand, SS-C composite particles allow the use of large amounts of sewage sludge and the inhibition of odor release from sludge; on the other hand, it also provides a basis for other researchers to apply the “multiscale composite particle preparation method” to other raw materials to adsorb or inhibit the release of certain gases. 

## Figures and Tables

**Table 1:** Proximate analysis of sewage sludge (wt.%).

M_a_	V_d_	A_d_	FC	Q_b,ad_ (MJ/kg)
78.1	41.64	52.46	5.91	15.09

M, moisture content; V, volatile content; A, ash content; FC, fixed carbon; Q_b_, refers to the bomb; calorific value. a, as received basis; d, dried basis; ad, refers to air-dried basis; daf, dried and ash-free basis.

**Table 2:** Chemical composition of sewage sludge (wt.%).

SiO_2_	Al_2_O_3_	Fe_2_O_3_	P_2_O_5_	CaO	K_2_O	MgO	SO_3_	Na_2_O	TiO_2_
37.076	20	14.99	12.076	5.792	2.543	2.336	2.215	1.038	0.843

**Table 3:** Chemical composition of clay (wt.%).

SiO_2_	CaO	Al_2_O_3_	Fe_2_O_3_	MgO	K_2_O	Na_2_O	TiO_2_	P_2_O_5_	MnO
37.66	19.87	10.2	4.733	2.746	2.02	0.693	0.595	0.206	0.0824

**Table 4:** Ratio and number of multiscale composite particles of sewage sludge-clay.

Number	1#	2#	3#	4#	5#	6#
SS (g):Clay (g)	1:5	1:10	1:15	1:20	1:25	1:30

**Table 5:** Internal structural changes in SS-C composite particles.

	NO.	1#	2#	3#	4#	5#	6#
T							
105 °C		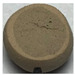	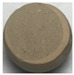	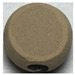	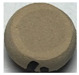	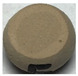	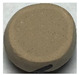
200 °C		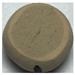	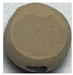	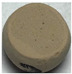	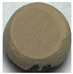	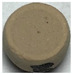	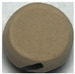
300 °C		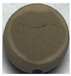	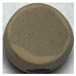	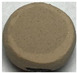	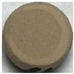	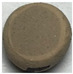	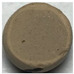
360 °C		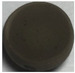	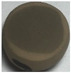	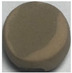	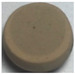	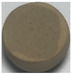	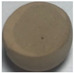
400 °C		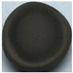	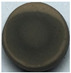	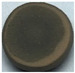	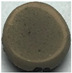	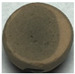	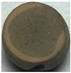
460 °C		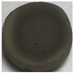	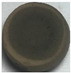	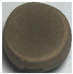	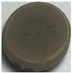	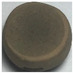	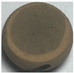
500 °C		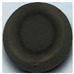	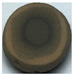	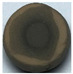	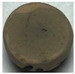	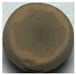	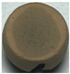
560 °C		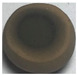	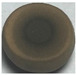	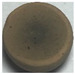	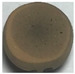	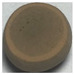	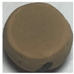
600 °C		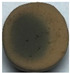	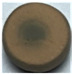	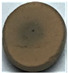	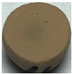	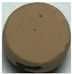	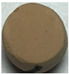
660 °C		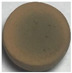	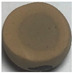	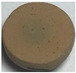	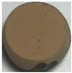	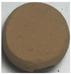	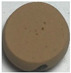
700 °C		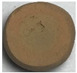	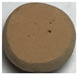	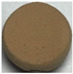	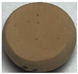	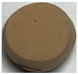	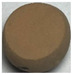
800 °C		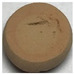	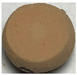	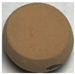	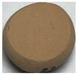	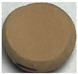	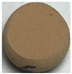
900 °C		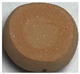	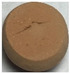	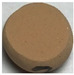	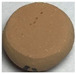	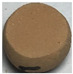	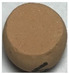
1000 °C		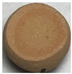	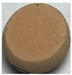	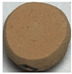	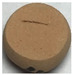	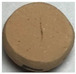	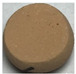
1100 °C		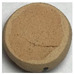	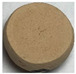	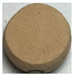	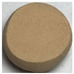	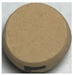	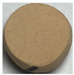

## Data Availability

Data available on request due to restrictions privacy. The data presented in this study are available on request from the corresponding author.
